# Simple Bayesian testing of scientific expectations in linear regression models

**DOI:** 10.3758/s13428-018-01196-9

**Published:** 2019-03-22

**Authors:** J. Mulder, A. Olsson-Collentine

**Affiliations:** 1 Department of Methodology and Statistics, Tilburg University, Warrandelaan 1, Tilburg, The Netherlands; 2 Jheronimus Academy of Data Science, Sint Janssingel 92, 5211 DA, ’s-Hertogenbosch, The Netherlands

**Keywords:** Bayes factors, Bayesian hypothesis testing, Equality and order constraints, Regression modeling

## Abstract

Scientific theories can often be formulated using equality and order constraints on the relative effects in a linear regression model. For example, it may be expected that the effect of the first predictor is larger than the effect of the second predictor, and the second predictor is expected to be larger than the third predictor. The goal is then to test such expectations against competing scientific expectations or theories. In this paper, a simple default Bayes factor test is proposed for testing multiple hypotheses with equality and order constraints on the effects of interest. The proposed testing criterion can be computed without requiring external prior information about the expected effects before observing the data. The method is implemented in R-package called ‘lmhyp’ which is freely downloadable and ready to use. The usability of the method and software is illustrated using empirical applications from the social and behavioral sciences.

## Introduction

The linear regression model is the most widely used statistical method for assessing the relative effects of a given set of predictors on a continuous outcome variable. This assessment of the relative effects is an essential part when testing, fine-graining, and building scientific theories. For example, in work and organizational psychology, the regression model has been used to better understand the effects of discrimination by coworkers and managers on workers’ well-being (Johnson et al., 2012); in sociology to assess the effects of the different dimensions of socioeconomic status on one’s attitude towards immigrants (Scheepers, Gijsberts, & Coenders, 2002); and in experimental psychology to make inferences regarding the effects of gender when hiring employees (Carlsson & Sinclair, 2017). Despite the extensive literature on statistical tools for linear regression analysis, methods for evaluating multiple hypotheses with equality and order constraints on the relative effects in a direct manner are still limited. This paper presents a Bayes factor testing procedure with accompanying software for testing such hypotheses with the goal of aiding researchers in the development and evaluation of scientific theories.

As an example, let us consider the following linear regression model where a dependent variable is regressed on three predictor variables, say, *X*_1_, *X*_2_, and *X*_3_:

$$ \begin{array}{@{}rcl@{}} y_{i} = \beta_{0} +\beta_{1} X_{i,1} + \beta_{2} X_{i,2} + \beta_{3} X_{i,3} + \epsilon_{i}, \end{array} $$

where *y*_*i*_ is the dependent variable of the *i*-th observation, *X*_*i*,*k*_ denotes the *k* predictor variable of the *i*-th observation, *β*_*k*_ is the regression coefficient of the *k*-th predictor, for *k* = 1,…,3, *β*_0_ is the intercept, and *𝜖*_*i*_ are independent normally distributed errors with unknown variance *σ*^2^, for *i* = 1,…,*n*.

In exploratory studies, the interest is typically whether each predictor has an effect on the dependent variable, and if there is evidence of a nonzero effect, we would be interested in whether the effect is positive or negative. In the proposed methodology, such an exploratory analysis can be executed by simultaneously testing whether an effect is zero, positive, or negative. For the first predictor, the exploratory multiple hypothesis test would be formulated as

1 $$ \begin{array}{@{}rcl@{}} H_{0}&:&\beta_{1}= 0 \\ H_{1}&:&\beta_{1}>0\\ H_{2}&:&\beta_{1}<0. \end{array} $$

The proposed Bayes factor test will then provide a default quantification of the relative evidence in the data between these hypotheses.

In confirmatory studies, the interest is typically in testing specific hypotheses with equality and order constraints on the relative effects based on scientific expectations or psychological theories (Hoijtink, 2011). Contrasting regression effects against each other using equality or order constraints can be more informative than interpreting them at certain benchmark values (e.g., standardized effects of .2, .5, and 1, are sometimes interpreted as ‘small’, ‘medium’, and ‘large’ effects, respectively) because effects are not absolute but relative quantifications; relative to each other and relative to the scientific field and context (Cohen, 1988). For example, a standardized effect of .4 may be important for an organizational psychologist who is interested in the effect of discrimination on well-being on the work floor but less so for a medical psychologist who wishes to predict the growth of a tumor of a patient through a cognitive test. As such, interpreting regression effects relative to each other using equality and order constraints would be more insightful than interpreting the effects using fixed benchmarks.

In the above regression model for instance, let us assume that *β*_1_, *β*_2_, and *β*_3_ denote the effects of a strong, medium, and mild treatment, respectively. It may then be hypothesized that the effect of the strong treatment is larger than the effect of the medium treatment, the effect of the medium treatment is expected to be larger than the effect of the mild treatment, and all effects are expected to be positive. Alternatively, it may be expected that all treatments have an equal positive effect. These hypotheses can then be tested against a third hypothesis which complements the other hypotheses. This comes down to the following multiple hypothesis test:

2 $$ \begin{array}{@{}rcl@{}} H_{1}&:&\beta_{1} > \beta_{2}>\beta_{3}>0 \\ H_{2}&:&\beta_{1} = \beta_{2}=\beta_{3}>0\\ H_{3}&:&\text{neither \(H_{1}\), nor \(H_{2}\)}. \end{array} $$

Here the complement hypothesis *H*_3_ covers the remaining possible values of *β*_1_, *β*_2_, and *β*_3_ that do not satisfy the constraints under *H*_1_ and *H*_2_. Subsequently, the interest is in quantifying the relative evidence in the data for these hypotheses.

A general advantage of Bayes factors for testing statistical hypotheses is that we obtain a direct quantification of the evidence in the data in favor of one hypothesis against another hypothesis. Furthermore, Bayes factors can be translated to the posterior probabilities of the hypotheses given the observed the data and the hypotheses of interest. These probabilities give a direct answer to the research question which hypothesis is most likely to be true and to what degree given the data. These posterior probabilities can be used to obtain conditional error probabilities of drawing an incorrect conclusion when ‘selecting’ a hypothesis in light of the observed data. These and other properties have greatly contributed to the increasing popularity of Bayes factors for testing hypotheses in psychological research (Mulder & Wagenmakers, 2016).

The proposed testing criterion is based on the prior adjusted default Bayes factor (Mulder, 2014b). The method has various attractive properties. First, the proposed Bayes factor has an analytic expression when testing hypotheses with equality and order constraints in a regression model. Thus, computationally demanding numerical approximations can be avoided, resulting in a fast and simple test. Furthermore, by allowing users to formulate hypotheses with equality as well as ordinal constraints, a broad class of hypotheses can be tested in an easy and direct manner. Another useful property is that no proper (subjective) prior distribution needs to be formulated based on external prior knowledge, and therefore the method can be applied in an automatic fashion. This is achieved by adopting a fractional Bayes methodology (O’Hagan, 1995) where a default prior is implicitly constructed using a minimal fraction of the information in the observed data and the remaining (maximal) fraction is used for hypothesis testing (Gilks, 1995). This default prior is then relocated to the boundary of the constrained space of the hypotheses. In the confirmatory test about the unconstrained default prior for (*β*_1_,*β*_3_,*β*_3_) would be centered around **0**. Because this Bayes factor can be computed without requiring external prior knowledge, it is called a ‘default Bayes factor’. Thereby, these default Bayes factors differ from regular Bayes factors where a proper prior is specified reflecting the anticipated effects based on external prior knowledge (e.g., Rouder & Morey, 2015). Other default Bayes factors that have been proposed in the literature are the fractional Bayes factor (O’Hagan, 1995), the intrinsic Bayes factor (Berger & Pericchi, 1996), and the Bayes factor based on expected-posterior priors (Pérez & Berger, 2002; Mulder et al., 2009).

Although various alternative testing procedures are available for hypothesis testing for linear regression analysis, these methods are limited to some degree. First, classical significance tests are only suitable for testing a null hypothesis against a single alternative, and unsuitable for testing multiple hypotheses with equality as well as order constraints (Silvapulle & Sen, 2004). Second, traditional model comparison tools (e.g., the AIC, BIC, or CFI) are generally not suitable for evaluating models (or hypotheses) with order constraints on certain parameters (Mulder et al., 2009; Braeken, Mulder, & Wood, 2015). Third, currently available Bayes factor tests cannot be used for testing order hypotheses (Rouder & Morey, 2015), are not computationally efficient (Mulder, Hoijtink, & de Leeuw, 2012; Kluytmans, van de Schoot, Mulder, & Hoijtink, 2012), or are based on large sample approximations (Gu, Mulder, & Hoijtink, 2018). The proposed Bayes factor, on the other hand, can be used for testing hypotheses with equality and/or order constraints, is very fast to compute due to its analytic expression, and is an accurate default quantification of the evidence in the data in the case of small to moderate samples because it does not rely on large sample approximations. Other important properties of the proposed methodology are its large sample consistent behavior and its information consistent behavior (Mulder, 2014b; Böing-Messing & Mulder, 2018).

The Bayesian test is implemented in the R-package ‘lmhyp’, which is freely downloadable and ready for use in R. The main function ‘test_hyp’ needs a fitted modeling object using the ‘lm’ function together with a string that formulates a set of hypotheses with equality and order constraints on the regression coefficients of interest. The function computes the Bayes factors of interest as well as the posterior probabilities that each hypothesis is true after observing the data.

The paper is organized as follows. Section “A default Bayes factor for equality and order hypotheses in a linear regression model” presents the derivation of the default Bayes factor between hypotheses with equality and order hypotheses on the relative effects in a linear regression model. Section “Software” presents the ‘lmhyp’ package and explains how it can be used for testing scientific expectations in psychological research. Section “Application of the new testing procedure using the software package ‘lmhyp’” shows how to apply the new procedure and software for testing scientific expectations in work and organizational psychology and social psychology. The paper ends with a short discussion.

## A default Bayes factor for equality and order hypotheses in a linear regression model

### Model and hypothesis formulation

For a linear regression model,

3 $$ \textbf{y}=\textbf{X}\boldsymbol{\beta}+N(\textbf{0},\sigma^{2}\textbf{I}_{n}), $$

where **y** is a vector of length *n* of outcome variables, **X** is a *n* × *k* matrix with the predictor variables, and ***β*** is a vector of length *k* containing the regression coefficients, consider a hypothesis with equality and inequality constraints on certain regression coefficients of the form

4 $$ H_{t}:\textbf{R}_{E}\boldsymbol{\beta}=\textbf{r}_{E} ~~~\&~~~ \textbf{R}_{I}\boldsymbol{\beta}>\textbf{r}_{I}, $$

where [**R**_*E*_|**r**_*E*_] and [**R**_*I*_|**r**_*I*_] are the augmented matrices with *q*_*E*_ and *q*_*I*_ rows that contain the coefficients of the equality and inequality constraints, respectively, and *k* + 1 columns. For example, for the regression model from the introduction, with ***β*** = (*β*_0_,*β*_1_,*β*_2_,*β*_3_)^′^, and the hypothesis *H*_1_ : *β*_1_ > *β*_2_ > *β*_3_ > 0 in Eq. 2, the augmented matrix of the inequalities is given by

$$ [\textbf{R}_{I}|\textbf{r}_{I}] = \left[ \begin{array}{cccc|c} 0 & 1 & -1 & 0 & 0\\ 0 & 0 & 1 & -1 & 0\\ 0 & 0 & 0 & 1 & 0 \end{array} \right] $$

and for the hypothesis *H*_2_ : *β*_1_ = *β*_2_ = *β*_3_ > 0, the augmented matrices are given by

$$ \begin{array}{@{}rcl@{}} [\textbf{R}_{E}|\textbf{r}_{E}] &=& \left[ \begin{array}{cccc|c} 0 & 1 & -1 & 0 & 0\\ 0 & 0 & 1 & -1 & 0 \end{array} \right]\\ \left[ \textbf{R}_{I}|\textbf{r}_{I}\right] &=& \left[ \begin{array}{cccc|c} 0 & 0 & 0 & 1 & 0 \end{array} \right] \end{array} $$

The prior adjusted default Bayes factor will be derived for a constrained hypothesis in Eq. 4 against an unconstrained alternative hypothesis, denoted by $H_{u}:\boldsymbol {\beta }\in \mathbb {R}^{k}$, with no constraints on the regression coefficients. First, we transform the regression coefficients as follows

5 $$ \boldsymbol{\xi}=\left[\begin{array}{c} \boldsymbol{\xi}_{E}\\ \boldsymbol{\xi}_{I} \end{array}\right]=\left[\begin{array}{c} \textbf{R}_{E}\\ \textbf{D} \end{array}\right]\boldsymbol{\beta}=\textbf{T}\boldsymbol{\beta}, $$

where **D** is a (*k* − *q*_*E*_) × *k* matrix consisting of the unique rows of $\textbf {I}_{k}-\textbf {R}_{E}^{\prime }(\textbf {R}_{E}\textbf {R}_{E})^{-1}\textbf {R}_{E}$. Thus, ***ξ***_*E*_ is a vector of length *q*_*E*_ and ***ξ***_*I*_ is a vector of length *k* − *q*_*E*_. Consequently, model (3) can be written as

$$ \textbf{y} = \textbf{X}\textbf{R}_{E}^{-1}\boldsymbol{\xi}_{E} + \textbf{X}\textbf{D}^{-1}\boldsymbol{\xi}_{I} + N(\textbf{0},\sigma^{2}\textbf{I}_{n}), $$

because

$$ \textbf{X}\boldsymbol{\beta} = \textbf{X}\textbf{T}^{-1}\boldsymbol{\xi} = \textbf{X}\left[\textbf{R}_{E}^{-1}~\textbf{D}^{-1}\right] \left[\begin{array}{c} \boldsymbol{\xi}_{E}\\ \boldsymbol{\xi}_{I} \end{array}\right] = \textbf{X}\textbf{R}_{E}^{-1}\boldsymbol{\xi}_{E}+\textbf{X}\textbf{D}^{-1}\boldsymbol{\xi}_{I}, $$

where $\textbf {R}_{E}^{-1}$ and **D**^− 1^ are the (Moore–Penrose) generalized inverse matrices of **R**_*E*_ and **D**, and the hypothesis in Eq. 4 can be written as

6 $$ H_{t}:\boldsymbol{\xi}_{E}=\textbf{r}_{E} ~~~\&~~~ \tilde{\textbf{R}}_{I}\boldsymbol{\xi}_{I}>\tilde{\textbf{r}}_{I}, $$

because

$$ \textbf{R}_{E}\boldsymbol{\beta} = \textbf{R}_{E}\textbf{T}^{-1}\boldsymbol{\xi} = \textbf{R}_{E}\left[\textbf{R}_{E}^{-1}~\textbf{D}^{-1}\right]\boldsymbol{\xi} = \left[\textbf{I}_{q_{E}}~\textbf{0}\right]\boldsymbol{\xi} = \boldsymbol{\xi}_{E}=\textbf{r}_{E} $$

and

$$ \begin{array}{@{}rcl@{}} &&\textbf{R}_{I}\boldsymbol{\beta} = \textbf{R}_{I}\textbf{T}^{-1}\boldsymbol{\xi} = \textbf{R}_{I}\left[\textbf{R}_{E}^{-1}~\textbf{D}^{-1}\right] \left[\begin{array}{c} \textbf{r}_{E}\\ \boldsymbol{\xi}_{I} \end{array}\right] = \textbf{R}_{I}\textbf{R}_{E}^{-1}\textbf{r}_{E}+\textbf{R}_{I}\textbf{D}^{-1}\boldsymbol{\xi}_{I}>\textbf{r}_{I}\\ &&\Leftrightarrow \tilde{\textbf{R}}_{I}\boldsymbol{\xi}_{I}>\tilde{\textbf{r}}_{I},~\text{with}~\tilde{\textbf{r}}_{I}=\textbf{r}_{I}-\textbf{R}_{I}\textbf{R}_{E}^{-1}\textbf{r}_{E}~\text{and}~\tilde{\textbf{R}}_{I}=\textbf{R}_{I}\textbf{D}^{-1}. \end{array} $$

### A default Bayes factor for testing hypotheses

The Bayes factor for hypothesis *H*_1_ against *H*_2_ is defined as the ratio of their respective marginal likelihoods,

$$ B_{12}=\frac{p_{1}(\textbf{y})}{p_{2}(\textbf{y})}. $$

The marginal likelihood quantifies the probability of the observed data under a hypothesis (Jeffreys, 1961; Kass & Raftery, 1995). For example, if *B*_12_ = 10 this implies that the data were ten times more likely to have been observed under *H*_1_ than under *H*_2_. Therefore, the Bayes factor can be seen as a relative measure of evidence in the data between two hypotheses. The marginal likelihood under a constrained hypothesis *H*_*t*_ in Eq. 4 is obtained by integrating the likelihood over the order constrained subspace of the free parameters weighted with the prior distribution,

7 $$ p_{t}(\textbf{y}) = \iint_{\textbf{R}_{I}\boldsymbol{\beta}>\textbf{r}_{I}} p_{t}(\textbf{y}|\boldsymbol{\beta},\sigma^{2})\pi_{t}(\boldsymbol{\beta},\sigma^{2})d\boldsymbol{\beta} d\sigma^{2}, $$

where *p*_*t*_(**y**|***β***,*σ*^2^) denotes the likelihood of the data under hypothesis *H*_*t*_ given the unknown model parameters, and *π*_*t*_ denotes the prior distribution of the free parameters under *H*_*t*_. The prior quantifies the plausibility of possible values that the model parameters can attain before observing the data.

Unlike in Bayesian estimation, the choice of the prior can have a large influence on the outcome of the Bayes factor. For this reason, ad hoc or arbitrary prior specification should be avoided when testing hypotheses using the Bayes factor. However, specifying a prior that accurately reflects one’s uncertainty about the model parameters before observing the data can be a time-consuming and difficult task (Berger, 2006). A complicating factor in the case of testing multiple, say, 3 or more, hypotheses, is that priors need to be carefully formulated for the free parameters under all hypotheses separately. Because noninformative improper priors also cannot be used when computing marginal likelihoods, there has been increasing interest in the development of default Bayes factors where ad hoc or subjective prior specification is avoided. In these default Bayes factors, a proper default prior is often (implicitly) constructed using a small part of the data while the remaining part is used for hypothesis testing. An example is the fractional Bayes factor (O’Hagan, 1995) where the marginal likelihood is defined by

8 $$ p_{t}(\textbf{y}) = \iint_{\textbf{R}_{I}\boldsymbol{\beta}>\textbf{r}_{I}} p_{t}(\textbf{y}|\boldsymbol{\beta},\sigma^{2})^{1-b}\pi_{t}(\boldsymbol{\beta},\sigma^{2}|\textbf{y}^{b})d\boldsymbol{\beta} d\sigma^{2}, $$

where the (subjective) proper prior in Eq. 7 is replaced by a proper default prior based on a (minimal) fraction “*b*” of the observed data,^1^ and the likelihood is raised to a power equal to the remaining fraction “1 − *b*”, which is used for hypothesis testing.

In this paper, an adjustment of fractional Bayes factor is considered where the default prior is centered on the boundary (or null value) of the constrained space. The motivation for this adjustment is twofold. First, when testing a precise hypothesis, say, *H*_0_ : *β* = 0 versus *H*_1_ : *β*≠ 0, Jeffreys argued that a default prior for *β* under *H*_1_ should be concentrated around the null value because, if the null would be false, the true effect would likely to be close to the null, otherwise there would be no point in testing *H*_0_. Second, when testing hypotheses with inequality or order constraints, the prior probability that the constraints hold serves as a measure of the relative complexity (or size) of the constrained space under a hypothesis (Mulder, Hoijtink, & Klugkist, 2010). This quantification of relative complexity of a hypothesis is important because the Bayes factor balances fit and complexity as an Occam’s razor. This implies that simpler hypotheses (i.e., hypotheses having “smaller” parameter spaces) would be preferred over more complex hypotheses in the case of an approximately equal fit. Only when centering the prior at 0 when testing *H*_1_ : *β* < 0 versus *H*_2_ : *β* > 0, both hypotheses would be considered as equally complex with prior probabilities of .5 corresponding to half of the complete parameter space of *β* of all real values ($\mathbb {R}$).

Given the above considerations, the fractional Bayes factor is adjusted such that the default prior is (i) centered on the boundary of the constrained parameter space and (ii) contains minimal information by specifying a minimal fraction. Because the model consists of *k* + 1 unknown parameters (*k* regression coefficients and an unknown error variance), a default prior is obtained using a minimal fraction^2^ of $b=\frac {k + 1}{n}$.

In order to satisfy the prior property (i) when testing a hypothesis (6), the prior for ***β*** under the alternative should thus be centered at **R**^− 1^**r**, where **R**^′^ = [**R***E*′ **R***I*′] and **r**^′^ = (**r***E*′,**r***I*′), which is equivalent to centering the prior for ***ξ*** at ***μ***^0^ = (***μ****E*0^′^,***μ****I*0^′^)^′^ = **TR**^− 1^**r** = (**r***E*′,***μ****I*0^′^ )^′^, with **R**~_*I*_***μ****I*0 = **r**~_*I*_. The following lemma gives the analytic expression of the default Bayes factor of a hypothesis with equality and order constraints on the regression coefficients versus an unconstrained alternative.

#### **Lemma 1**

*The prior adjusted default Bayes factors for an equality-constrained hypothesis,H*_1_ : **R**_*E*_***β*** = **r**_*E*_*,an order-constrained hypothesis,H*_2_ : **R**_*I*_***β*** > **r**_*I*_*,and a hypothesis with equality and order constraints,H*_3_ : **R**_*E*_***β*** = **r**_*E*_*,***R**_*I*_***β*** > **r**_*I*_*,against an unconstrained hypothesis*$H_{u}:\boldsymbol {\beta }\in \mathbb {R}^{k}$*aregiven by*

9 $$ \begin{array}{@{}rcl@{}} B_{1u}\!&=&\!\! \frac{{f^{E}_{1}}}{{c^{E}_{1}}}=\frac{t(\textbf{r}_{E};\textbf{R}_{E}\hat{\boldsymbol{\beta}},s^{2}(n-k)^{-1}\textbf{R}_{E}(\textbf{X}^{\prime}\textbf{X})^{-1}\textbf{R}_{E}^{\prime},n-k)} {t(\textbf{r}_{E};\textbf{r}_{E},s^{2}\textbf{R}_{E}(\textbf{X}^{\prime}\textbf{X})^{-1}\textbf{R}_{E}^{\prime},1)}, \end{array} $$

10 $$ \begin{array}{@{}rcl@{}} B_{2u}\!&=&\!\!\frac{{f^{I}_{2}}}{{c^{I}_{2}}}=\frac{\text{Pr}(\textbf{R}_{I}\boldsymbol{\beta}>\textbf{r}_{I}|\textbf{y},H_{u})}{\text{Pr}(\textbf{R}_{I}\boldsymbol{\beta}>\textbf{r}_{I}|\textbf{y}^{b},H_{u})}, \end{array} $$

11 $$ \begin{array}{@{}rcl@{}} B_{3u}\!&=&\!\!\frac{{f^{E}_{3}}}{{c^{E}_{3}}} \!\times\! \frac{f^{I|E}_{3}}{c^{I|E}_{3}} = \frac{t(\textbf{r}_{E};\textbf{R}_{E}\hat{\boldsymbol{\beta}},s^{2}(n - k)^{-1}\textbf{R}_{E}(\textbf{X}^{\prime}\textbf{X})^{-1}\textbf{R}_{E}^{\prime},n - k)} {t(\textbf{r}_{E};\textbf{r}_{E},s^{2}\textbf{R}_{E}(\textbf{X}^{\prime}\textbf{X})^{-1}\textbf{R}_{E}^{\prime},1)}\\ \end{array} $$

12 $$ \begin{array}{@{}rcl@{}} &&\times \frac{\text{Pr}(\tilde{\textbf{R}}_{I}\boldsymbol{\xi}_{I}>\tilde{\textbf{r}}_{I}|\boldsymbol{\xi}_{E}=\textbf{r}_{E},\textbf{y},H_{u})}{\text{Pr}(\tilde{\textbf{R}}_{I}\boldsymbol{\xi}_{I}>\textbf{r}^{*}_{I}|\boldsymbol{\xi}_{E}=\hat{\boldsymbol{\xi}}_{E},\textbf{y}^{b},H_{u})}, \end{array} $$

where $\textbf {r}^{*}_{I}=\tilde {\textbf {R}}_{I}\hat {\boldsymbol {\xi }}_{I}$, *t*(***ξ***;***μ***,**S**,*ν*) denotes a Student’s *t* density for ***ξ*** with location parameter ***μ***, scale matrix **S**, and degrees of freedom *ν*, $\hat {\boldsymbol {\beta }}=(\textbf {X}^{\prime }\textbf {X})^{-1}\textbf {X}^{\prime }\textbf {y}$ is the maximum likelihood estimate (MLE) of ***β*** and $s^{2}=(\textbf {y}-\textbf {X}\hat {\boldsymbol {\beta }})^{\prime }(\textbf {y}-\textbf {X}\hat {\boldsymbol {\beta }})$ is the sums of squares, and the (conditional) distributions are given by

$$ \begin{array}{@{}rcl@{}} \pi(\boldsymbol{\beta}|\textbf{y},H_{u})& = &t(\boldsymbol{\beta};\hat{\boldsymbol{\beta}},s^{2}(\textbf{X}^{\prime}\textbf{X})^{-1}/(n - k),n - k)\\ \pi(\boldsymbol{\beta}|\textbf{y}^{b},H_{u})& = &t(\boldsymbol{\beta};\textbf{R}^{-1}_{I}\textbf{r}_{I},s^{2}(\textbf{X}^{\prime}\textbf{X})^{-1},1)\\ \pi(\boldsymbol{\xi}_{I}|\boldsymbol{\xi}_{E} = \textbf{r}_{E},\textbf{y},H_{u})& = &t(\boldsymbol{\xi}_{I};{\boldsymbol{\mu}_{I}^{N}},\textbf{S}_{I}^{N},n-k)\\ \pi(\boldsymbol{\xi}_{I}|\boldsymbol{\xi}_{E} = \textbf{r}_{E},\textbf{y}^{b},H_{u})& = &t(\boldsymbol{\xi}_{I};{\boldsymbol{\mu}_{I}^{0}},\textbf{S}_{I}^{0},1) \end{array} $$

with

$$ \begin{array}{@{}rcl@{}} {\boldsymbol{\mu}_{I}^{N}}& = &\textbf{D}\hat{\boldsymbol{\beta}}+\textbf{D}(\textbf{X}^{\prime}\textbf{X})^{-1}\textbf{R}_{E}^{\prime}(\textbf{R}_{E}(\textbf{X}^{\prime}\textbf{X})^{-1}\textbf{R}_{E}^{\prime})^{-1}(\textbf{r}_{E}-\textbf{R}_{E}\hat{\boldsymbol{\beta}})\\ \textbf{S}_{I}^{N}& = &\left( 1 + s^{-2}(\textbf{r}_{E} - \textbf{R}_{E}\hat{\boldsymbol{\beta}})'(\textbf{R}_{E}(\textbf{X}^{\prime}\textbf{X})^{-1}\textbf{R}_{E}^{\prime})^{-1}(\textbf{r}_{E} - \textbf{R}_{E}\hat{\boldsymbol{\beta}})\right)(n - k + q_{E})^{-1}s^{2}\\ &&(\textbf{D}(\textbf{X}^{\prime}\textbf{X})^{-1}\textbf{D}^{\prime} - \textbf{D}(\textbf{X}^{\prime}\textbf{X})^{-1}\textbf{R}_{E}^{\prime}(\textbf{R}_{E}(\textbf{X}^{\prime}\textbf{X})^{-1}\textbf{R}_{E}^{\prime})^{-1}\textbf{R}_{E}(\textbf{X}^{\prime}\textbf{X})^{-1}\textbf{D}^{\prime})\\ \textbf{S}_{I}^{0}& = &\tfrac{s^{2}}{1+q^{E}}(\textbf{D}(\textbf{X}^{\prime}\textbf{X})^{-1}\textbf{D}^{\prime} - \textbf{D}(\textbf{X}^{\prime}\textbf{X})^{-1}\textbf{R}_{E}^{\prime}(\textbf{R}_{E}(\textbf{X}^{\prime}\textbf{X})^{-1}\textbf{R}_{E}^{\prime})^{-1}\textbf{R}_{E}(\textbf{X}^{\prime}\textbf{X})^{-1}\textbf{D}^{\prime}), \end{array} $$

#### Proof

Appendix A.

Note that the factors in Eqs. 9 and 12 are multivariate Savage–Dickey density ratio’s (Dickey 1971; Wetzels, Grasman, & Wagenmakers, 2010; Mulder et al., 2010). These ratios have an analytic expression because the marginal posterior and default prior have multivariate Student’s t distributions. In R, these can be computed using the dmvt function in the mvtnorm-package (Genz et al., 2016).

The ratios of (conditional) probabilities in Eqs. 10 and 12 can also be computed in a straightforward manner. If $\tilde {\textbf {R}}_{I}$ is of full row-rank, then the transformed parameter vector, say, $\boldsymbol {\eta }_{I}=\tilde {\textbf {R}}_{I}\boldsymbol {\xi }_{I}$ has a Student’s t distribution so that $\text {Pr}(\tilde {\textbf {R}}_{I}\boldsymbol {\xi }_{I}>\tilde {\textbf {r}}_{I}|\boldsymbol {\xi }_{E}=\textbf {r}_{E},\textbf {y},H_{u})=\text {Pr}(\boldsymbol {\eta }_{I}>\tilde {\textbf {r}}_{I}|\boldsymbol {\xi }_{E}=\textbf {r}_{E},\textbf {y},H_{u})$ can be computed using the pmvt function from the mvtnorm-package (Genz et al., 2016). If the rank of $\tilde {\textbf {R}}_{I}$ is lower than *q*^*I*^, then the probability can be computed as the proportion of draws from an unconstrained Student’s t distribution satisfying the order constraints.

The posterior quantities in the numerators reflect the relative fit of a constrained hypothesis, denoted by “f ”, relative to the unconstrained hypothesis: a larger posterior probability implies a good fit of the order constraints and a large posterior density at the null value indicates a good fit of a precise hypothesis. The prior quantities in the denominators reflect the relative complexity of a constrained hypothesis, denoted by “c”, relative to the unconstrained hypothesis: a small prior probability implies a relatively small inequality constrained subspace, and thus a ‘simple’ hypothesis, and a small prior density at the null value corresponds to a large spread (variance) of possible values under the unconstrained alternative implying the null hypothesis is relatively simple in comparison to the unconstrained hypothesis.

**Fig. 1:**
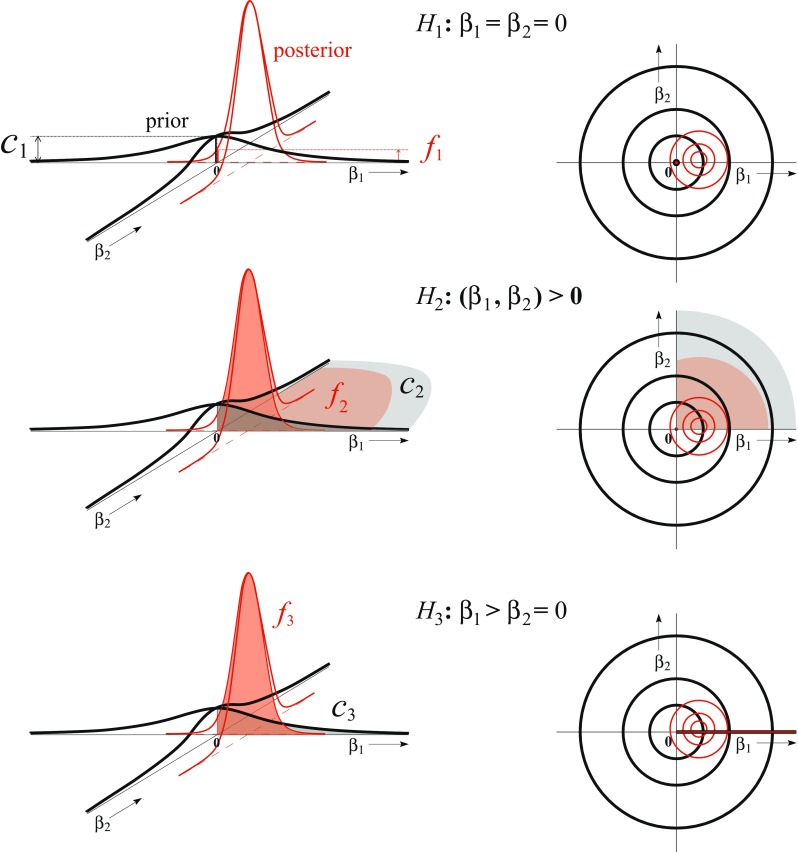
Graphical representation of the default Bayes factor for *H*_1_ : *β*_1_ = *β*_2_ = 0 (upper panels), *H*_2_ : ***β*** > **0** (middle panels), and *H*_3_ : *β*_1_ > *β*_2_ = 0 (lower panels) as the ratios of the posterior (red thin lines) and prior (black thick lines) density at the null value, the posterior and prior probabilities, and the surfaces of the cross sections of the posterior and prior density, respectively

Figure 1 gives more insight about the nature of the expressions in Eqs. 9 to 12 in Lemma 1 for an equality constrained hypothesis, *H*_1_ : *β*_1_ = *β*_2_ = 0 (upper panels), an inequality constrained hypothesis, *H*_2_ : ***β*** > **0** (middle panels), and hypothesis with an equality constraint and an inequality constraint, *H*_3_ : *β*_1_ > *β*_2_ = 0 (lower panels). The Bayes factor for *H*_1_ against the unconstrained hypothesis *H*_*u*_ in Eq. 9 corresponds to the ratio of the unconstrained posterior density and the unconstrained default prior (which has a multivariate Cauchy distribution centered at the null value) evaluated at the null value. The Bayes factor for *H*_2_ against *H*_*u*_ in Eq. 10 corresponds to the ratio of posterior and default prior probabilities that the constraints hold under *H*_*u*_. In the case of independent predictors, for example, the prior probability would be equal .25 as a result of centering the default prior at **0**. The inequality constrained hypothesis would then be quantified as four times less complex than the unconstrained hypothesis. Finally, for a hypothesis with equality and inequality constraints, *H*_3_ : *β*_1_ > *β*_2_ = 0, the Bayes factor in Eqs. 11–12 corresponds to the ratio of the surfaces of cross section of the posterior and prior density on the line *β*_1_ > 0, *β*_2_ = 0.

The default Bayes factors between these hypotheses are computed for a simulated data set with MLEs $(\hat {\beta }_{1},\hat {\beta }_{2})=(.7,.03)$ (Appendix B) that results in *B*_1*u*_ = *f*1*Ec*1*E* = 0.061 0.159 = 0.383,*B*_2*u*_ = *f*2*Ic*2*I* = 0.546 0.250 = 2.183,*B*_3*u*_ = *f*3*Ec*3*E* ×*f*3*I*|*Ec*3*I*|*E* = 1.608 0.318 ×0.996 0.500 = 10.061. As will be explained in the next section, it is recommendable to include the complement hypothesis in an analysis. The complement hypothesis covers the subspace of $\mathbb {R}^{2}$ that excludes the subspaces under *H*_1_, *H*_2_, and *H*_3_. In this example, the Bayes factor of the complement hypothesis against the unconstrained hypothesis equals $B_{cu}=\frac {1-{f^{I}_{2}}}{1-{c^{I}_{2}}}=\frac {0.454}{0.750}= 0.606$.

After having obtained the default Bayes factor of each hypothesis against the unconstrained hypothesis, Bayes factors between the hypotheses of interest can be obtained through the transitivity property of the Bayes factor, e.g., $B_{31}=\frac {B_{3u}}{B_{1u}}=\frac {10.061}{.383}= 26.299$. This implies that there is strong evidence for *H*_3_ relative to *H*_1_, as the data were approximately 26 times more likely to have been produced under *H*_3_ than under *H*_1_.

Once the default Bayes factors of the hypotheses of interest against the unconstrained hypothesis are computed using Lemma 1, posterior probabilities can be computed for the hypotheses. In the case of, say, four hypotheses of interest against, the posterior probability that hypothesis *H*_*t*_ is true can be obtained via

13 $$ \text{Pr}(H_{t}|\textbf{y})= \frac{B_{tu}\text{Pr}(H_{t})}{B_{1u}\text{Pr}(H_{1})+B_{2u}\text{Pr}(H_{2})+B_{3u}\text{Pr}(H_{3}) +B_{cu}\text{Pr}(H_{c})}, $$

for *t* = 1, 2, or 3, where Pr(*H*_*t*_) denotes the prior probability of hypothesis *H*_*t*_, i.e., the probability that *H*_*t*_ is true before observing the data. As can be seen, the posterior probability is a weighted average of the Bayes factors weighted with the prior probabilities. Throughout this paper, we will work with equal prior probabilities, but other choices may be preferred in specific applications (e.g., Wagenmakers, Wetzels, Borsboom, & van der Maas, 2011). For the example data from Appendix B and Fig. 1, the posterior probabilities would be equal to *P*(*H*_1_|**y**) = 0.029, *P*(*H*_2_|**y**) = 0.165, *P*(*H*_3_|**y**) = 0.760, and *P*(*H*_*c*_|**y**) = 0.046. Based on these outcomes, we would conclude that there is most evidence for *H*_3_ that the effect of the first predictor is positive and the effect of the second predictor is zero with a posterior probability of .76. In order to draw a more decisive conclusion (e.g., when obtaining a posterior probability for a hypothesis larger than, say, .99) more data are needed. □

## Software

The Bayes factor testing criterion for evaluating equality and order-constrained hypotheses was implemented in a new R package called ‘lmhyp’ to ensure general utilization of the methodology.^3^ As input, the main function ‘test_hyp’ needs a fitted linear regression modeling object from the lm-function as well as a string that specifies the constrained hypotheses of interest.

As output, the function provides the default Bayes factors between all pairs of hypotheses. By default a complement hypothesis is also included in the analysis. For example, when testing the hypotheses, say, *H*_1_ : *β*_1_ > *β*_2_ > *β*_3_ > 0 versus *H*_2_ : *β*_1_ = *β*_2_ = *β*_3_ > 0, a third complement hypothesis *H*_3_ will be automatically added, which covers the remaining parameter space, i.e., $\mathbb {R}^{3}$ excluding the subspaces under *H*_1_ and *H*_2_. The reason for including the complement hypothesis is that Bayes factors provide relative measures of evidence between the hypotheses. For example, it may be that *H*_2_ receives, say, 30 times more evidence than *H*_1_, i.e., *B*_21_ = 30, which could be seen as strong evidence for *H*_2_ relative to *H*_1_, yet it may be that *H*_2_ still badly fits to the data in an absolute sense. In this case, the evidence for the complement hypothesis *H*_3_ against *H*_2_ could be very large, say, *B*_32_ = 100.

Besides the default Bayes factor, the function also provides the posterior probabilities of the hypotheses. Posterior probabilities may be easier for users to interpret than Bayes factors because the posterior probabilities sum up to 1. Note that when setting equal prior probabilities between two hypotheses, the posterior odds of the hypotheses will be equal to the Bayes factor. By default, all hypotheses receive equal prior probabilities. Thus, in the case of *T* hypotheses, then $P(H_{t})=\frac {1}{T}$, for *t* = 1,…,*T*. Users can manually specify the prior probabilities by using the ‘priorprobs’ argument. In the remaining part of the paper, we will work with the default setting of equal prior probabilities. A step-by-step guide for using the software will be provided in the following section.

## Application of the new testing procedure using the software package ‘lmhyp’

In this section, we illustrate how to use the ‘lmhyp’ package to test hypotheses, by applying the procedure to two empirical examples from psychology. We begin by describing the published research. In the two following subsections, we then formulate hypotheses for each example and test these using the function test_hyp from our R-package lmhyp.^4^

For the first example, we use data from a study of mental health workers in England (Johnson et al., 2012). The data of Johnson et al. measured health workers’ well-being and its correlates, such as perceived discrimination from managers, coworkers, patients, and visitors. Well-being was operationalized by scales measuring anxiety, depression, and job dissatisfaction, the first two scales consisting of three items and the latter of five. The perceived discrimination variables are binary variables that were meant to capture whether the worker believed they had been discriminated against from the four different sources in the last 12 months. This example demonstrates hypothesis testing in regards to single variables and the “exploratory” option of the test_hyp function.

Our second empirical example comes from research by Carlsson and Sinclair (2017). Over four experiments, Carlsson and Sinclair compare two theoretical explanations for perceptions of gender discrimination in hiring, although we use data from only the first experiment (available at https://osf.io/qcdgp/). In this study, Carlsson and Sinclair showed university students two fictive job applications from a man and a woman for a position as either a computer specialist or nurse. Participants were told that the fictive job applications had been sent to real companies as part of a previous study, but that only one of the two applicants had been invited to a job interview despite being equally qualified. A two-item scale was then used to measure participants’ belief the outcome was due to gender discrimination. Several potential correlates were also measured using two-item scales, such as the individual’s belief that (wo)men are generally discriminated against, their expectation that they are gender-stereotyped by others (‘stigma consciousness’) and the extent to which they identify as feminists. This example demonstrates testing hypotheses involving multiple variables.

### Hypothesis testing of single effects in organizational psychology

In our first example, we illustrate how our approach might be used to explore competing hypotheses for single variables. It is common when testing the effect of an independent variable in regression to look at whether it is significantly different from zero, or to do a one-sided test of a positive versus a negative effect. When using a Bayes factor test, we can test all these hypotheses directly against each other and compare the relative evidence for each hypothesis.

Braeken et al., (2015) theorized that workplace discrimination has a negative impact on workers’ well-being. Here, we are testing this expectation against a positive effect and a zero effect, while controlling for discrimination from different sources. For example, in the case of discrimination by managers we have

14 $$ \begin{array}{l} H_{1}: \beta_{manager} < 0 \\ H_{2}: \beta_{manager} = 0 \\ H_{3}: \beta_{manager} > 0, \end{array} $$

while controlling for discrimination by coworkers, patients, and visitors through the following regression model

$$ \begin{array}{@{}rcl@{}} y_{anxiety,i} \!&=&\! \beta_{0} + \beta_{manager} X_{manager,i} + \beta_{coworkers} X_{coworker,i}\\ && + \beta_{patient} X_{patient,i} + \beta_{visitor} X_{visitor,i} + \text{error}_{i} \end{array} $$

where the *β*’s are the regression effects of the various sources of discrimination on anxiety.

Evaluating these three hypotheses in R is straightforward with the test_hyp function from our R-package lmhyp. This function takes as arguments ‘object’, a fitted object using the lm function, ‘hyp’, a string vector specifying one or several hypotheses (separated by semicolons), ‘priorprob’, specifying the prior probabilities of each hypotheses (by default equal, priorprob = 1), and ‘mcrep’, an integer that specifies the number of draws to compute the prior and posterior probabilities in the (unusual) case the matrix with the coefficients of the order constraints is not of full row rank (by default mcrep= 1e6). In addition, the argument hyp also allows as input the string ”exploratory”, which will test the likelihood of the data for a zero, positive, or negative effect of all variables in the regression model, including the intercept. We will make use of this functionality below, after first discussing how to test the three hypotheses for a single variable. To test the hypotheses, we first fit a linear model on the variables as usual:

fit <- lm(anx ~ discM + discC + discP+ discV, data = dat1)

Next, hypotheses are specified in R as character strings using the variable names from the fitted linear model. It is possible to test the traditional null hypothesis of *β*_*manager*_ = 0 against the two-sided alternative example *β*_*manager*_≠ 0 by writing

H2 <- "discM = 0"

Note that the complement hypothesis, *β*_*manager*_≠ 0, is automatically included. However, by testing whether the effect is zero, positive, or negative simultaneously, we obtain a more complete picture of the possible existence and direction of the population effect. This can be achieved by specifying all hypotheses as a single character vector in which the hypotheses are separated by semicolons:

Hyp1v2v3 <- "discM < 0; discM = 0; discM > 0"

Note that spacing does not matter. Once the hypotheses have been specified, they are tested by simply inputting them together with the fitted linear model object into the function test_hyp:

result <- test_hyp(fit, Hyp1v2v3)

This will compute the default Bayes factors from Lemma 1 between the hypotheses, as well as the posterior probabilities for the hypotheses. The posterior probabilities are printed as the primary output:

## Hypotheses:#### H1: "discM<0"## H2: "discM=0"## H3: "discM>0"#### Posterior probability of each hypothesis (rounded):#### H1: 0.000## H2: 0.000## H3: 1.000

As can be seen, the evidence is overwhelmingly in favor of a positive effect of discrimination from managers on anxiety amongst health workers. In fact, when concluding that *H*_3_ : *β*_*manager*_ > 0 is true, we would have a conditional error probability of drawing the wrong conclusion of approximately zero. To perform this test for all regression effects, one simply needs to set the second hyp argument equal to "exploratory":

result <- test_hyp(fit, "exploratory")

This option assumes that each hypothesis is equally likely *a priori*. In the current example, we then get the following output:

## Hypotheses:#### H1: "X < 0"## H2: "X = 0"## H3: "X > 0"#### Posterior probabilities for each variable (rounded),## assuming equal prior probabilities:#### H1 H2 H3## X < 0 X = 0 X > 0## (Intercept) 0.000 0.000 1.000## discM 0.000 0.000 1.000## discC 0.005 0.780 0.216## discP 0.003 0.628 0.369## discV 0.007 0.911 0.082

The posterior probabilities for discrimination by managers are the same as when tested separately. In regards to the other variables, there seems to be positive evidence that there is no effect of discrimination by coworkers, patients, or visitors on anxiety. Note that the evidence for this is not as compelling as for the effect of discrimination by managers, as can be seen from the conditional error probabilities of .216, .369, and .082, respectively, which are quite large. Therefore more data are needed in order to draw more decisive conclusions. Note here that classical significance tests cannot be used for quantifying the evidence in the data in favor of the null; the classical test can only be used to falsify the null. When a null hypothesis cannot be rejected, we are left in a state of ignorance because we cannot reject the null but also not claim there is evidence for the null (Wagenmakers, 2007).

Because the prior probabilities of the hypotheses are equal, the ratio of the posterior probabilities of two hypotheses corresponds with the Bayes factor, e.g., $B_{23}=\frac {\text {Pr}(H_{2}|\textbf {y})}{\text {Pr}(H_{3}|\textbf {y})}=\frac {.780}{.216}= 3.615$, for the effect of discrimination by coworkers. By calling BF_matrix, we obtain the default Bayes factors between all pairs of hypotheses. For convenience, the printed Bayes factors are rounded to three digits, though exact values can be calculated from the posterior probabilities (unrounded posterior probabilities are available by calling result$post_prob). The Bayes factor matrix for discC (discrimination from coworkers) can be obtained by calling

result\(BF_matrix\)discC## H1 H2 H3## H1 1.000 0.006 0.022## H2 162.367 1.000 3.615## H3 44.913 0.277 1.000

Hence, the null hypothesis of no effect is 162 times more likely than hypothesis *H*_1_ which assumes a negative effect (*B*_21_ = 162.367), but only 3.6 times more likely than hypothesis *H*_3_, which assumes a positive effect (*B*_23_ = 3.615). Similar Bayes factor matrices can be printed for all variables when using the “exploratory” option.

To summarize the first application, regressing the effects of perceived discrimination from managers, coworkers, patients, and visitors on the anxiety levels of English health workers, we found very strong evidence for a positive effect of perceived discrimination from managers on anxiety, mild-to-moderate evidence for no effect of discrimination from coworkers, patients, and visitors on anxiety. More research is needed to draw clearer conclusions regarding the existence of a zero or positive effect of these latter three variables.

### Hypothesis testing of multiple effects in social psychology

In our second example, we illustrate how our testing procedure can be used when testing multiple hypotheses with competing equal and order constraints on the effect of different predictor variables. Carlsson and Sinclair (2017) compared two different theoretical explanations for perceptions of gender discrimination in hiring for the roles of computer specialist and nurse. To test individual differences, they regressed perceptions of discrimination towards female victims on belief in discrimination against women, stigma consciousness, and feminist identification, while controlling for gender and belief in discrimination against men. As a regression equation, this can be expressed as

$$ \begin{array}{@{}rcl@{}} y_{discriminationW,i}& =& \beta_{0} + \beta_{beliefW} X_{beliefW,i} \\&&+ \beta_{stigma} X_{stigma,i} \\&&+ \beta_{feminist} X_{feminist} \\ &&+ \beta_{gender} X_{gender,i} \\ &&+ \beta_{beliefM} X_{beliefM,i} + \text{error}_{i}. \end{array} $$

where the *β*’s are standardized regression effects of the variables on perceived discrimination. Since in this subsection we will compare the beta-coefficients of different variables against each other, it facilitates interpretation if they are on the same scale. As such, we standardize all variables before entering them in the model.

The two theories that Carlsson and Sinclair (2017) examined make different explanations for what individual characteristics are most important to perceptions of gender discrimination. The ‘prototype explanation’ suggests that what matters are the individual’s beliefs that the gender in question is discriminated against, whereas the ‘same-gender bias explanation’ suggests that identification with the victim is most important. In our example, the victim of discrimination is female and Carlson and Sinclair operationalize identification with the victim as stigma consciousness and feminist identity. Note that neither theory makes any predictions regarding the control variables (gender and general belief that men are discriminated against). A first hypothesis, based on the prototype explanation, might thus be that belief in discrimination of women in general is positively associated with the belief that the female applicant has been discriminated against, whereas stigma consciousness and feminist identity have no effect on this belief. Formally, this can be expressed as

15 $$ H_{1}: \beta_{beliefW} > \beta_{stigma}= \beta_{feminist} = 0 $$

which is equivalent to:

16 $$ H_{1}: \beta_{beliefW} > (\beta_{stigma}, \beta_{feminist}) = 0 $$

Alternatively, we might expect all three variables to have a positive effect on the dependent variable (all *β*’s > 0), but that, in accordance with the prototype explanation, a belief that women are generally discriminated against should have a larger effect on perceptions of discrimination than identifying with the job applicant. Formally this implies:

17 $$ H_{2}: \beta_{beliefW} > (\beta_{stigma}, \beta_{feminist}) > 0 $$

A third hypothesis, based on the same-gender bias explanation, would be the reverse of the *H*_1_, namely that stigma consciousness and feminist identity are positively associated with the outcome while a general belief in discrimination against women has no impact on the particular case. That is:

18 $$ H_{3}: (\beta_{stigma}, \beta_{feminist}) > \beta_{beliefW} = 0 $$

In this example, we have thus specified three contradicting hypotheses regarding the relationships between three variables and wish to know which hypothesis receives most support from the data at hand. However, there is one additional implied hypothesis in this case: the complement. The complement, *H*_*c*_, is the hypothesis that none of the specified hypotheses are true. The complement exists if the specified hypotheses are not exhaustive, that is, do not cover the entire parameter space. In other words, the complement exists if there are possible values for the regression coefficients, which are not contained in the hypotheses, for example, (*β*_1_,*β*_2_,*β*_3_) = (− 1,− 1,− 1) is a combination of effects which do not satisfy the constraints of either *H*_1_, *H*_2_, or *H*_3_. Thus, the interest is in testing the following hypotheses:

$$ \begin{array}{@{}rcl@{}} H_{1}&:& \beta_{beliefW} > (\beta_{stigma}, \beta_{feminist}) = 0 \\ H_{2}&:& \beta_{beliefW} > (\beta_{stigma}, \beta_{feminist}) > 0 \\ H_{3}&:& (\beta_{stigma}, \beta_{feminist}) > \beta_{beliefW} = 0 \\ H_{c}&:& \text{not }H_{1}, H_{2}, H_{3} \end{array} $$

As before, we begin by fitting a linear regression on the (standardized) variables:

fit <- lm(discW ~ beliefW + stigma+ feminist+ beliefM + gender, data = dat2)

Next, we specify the hypotheses separated by semicolons as a character vector, here on separate lines for space reasons:

hyp1v2v3 <- "beliefW > (stigma,feminist)= 0;beliefW > (stigma, feminist) > 0;(stigma, feminist) > beliefW = 0"

The complement does not need to be specified, as the function will include it automatically if necessary. For this example, we get the following output:

## Hypotheses:#### H1: "beliefW>(stigma,feminist)=0"## H2: "beliefW>(stigma,feminist)>0"## H3: "(stigma,feminist)>beliefW=0"## Hc: "Not H1-H3"#### Posterior probability of each hypothesis (rounded):#### H1: 0.637## H2: 0.359## H3: 0.000## Hc: 0.004

From the output posterior probabilities, we see that *H*_1_ and *H*_2_, both based on the prototype explanation, received the most support, whereas *H*_3_, which was derived from the same-gender bias model, and the complementary hypothesis are both highly unlikely. These results can be succinctly reported as: “Using a default Bayes factor approach, we obtain overwhelming evidence that either hypothesis *H*_1_ or *H*_2_ is true with posterior probabilities of approximately .637, .359, .000, and .004 for *H*_1_, *H*_2_, *H*_3_, and *H*_4_, respectively.” Printing the Bayes Factor matrix yields:

result$BF_{m}atrix## H1 H2 H3 Hc## H1 1.000 1.776 1634.299 163.201## H2 0.563 1.000 920.205 91.892## H3 0.001 0.001 1.000 0.100## Hc 0.006 0.011 10.014 1.000

We see that the evidence for both *H*_1_ and *H*_2_ is very strong compared to the complement and in particular compared to *H*_3_, but that *H*_1_ is only 1.8 times as likely as *H*_2_ (*B*_12_ = 1.777).

To summarize the second application, our data demonstrated strong evidence for the prototype explanation and a lack of support for the same-gender bias explanation in explaining perceptions of discrimination against female applicants in the hiring process of computer specialist and nurses. The relative evidence for the prototype explanation depended on its exact formulation, but was at least 919 times stronger than for the same-gender bias explanation, and 91 times stronger than for the complement. However, further research is required to determine whether identification with a female victim has zero or a positive effect on perceived discrimination.

### Supplementary output

When saving results from the test_hyp function to an object it is possible to print additional supplementary output. This output is provided to support a deeper understanding of the method and the primary output outlined in the above subsections. We illustrate these two additional commands using the example in Section “Hypothesis testing of multiple effects in social psychology”. Calling BF_computation prints the measures of relative fit “*f* ” and complexity “*c*” in Eqs. 9 – 12 of the Bayes factor of each hypothesis against the unconstrained hypothesis. Thus, for the data and hypotheses of “Hypothesis testing of multiple effects in social psychology” we get

result$BF_computation## c(E) c(I|E) c f(E) f(I|E) f B(t,u) PP(t)## H1 0.151 0.500 0.075 4.398 1.000 4.398 58.265 0.639## H2 NA 0.020 NA NA 0.650 NA 32.525 0.357## H3 0.273 0.201 0.055 0.002 0.985 0.002 0.036 0.000## Hc NA 0.980 NA NA 0.350 NA 0.357 0.004

where c(E) is the prior density at the null value, c(I|E) the prior probability that the constraints hold, c the product of these two, and the columns labeled as f(E), f(I|E), and f have similar interpretations for the posterior quantities. B(t,u) is the Bayes factor of hypothesis *H*_*t*_ against the unconstrained (*H*_*u*_) and PP(t) is the posterior probability of hypothesis *H*_*t*_. We rounded the output to three decimals for convenience. Cells with “NA” indicate that a column is “Not Available” to a particular hypothesis. For example, because *H*_2_ contains only inequality comparisons it has a prior (and posterior) probability but no prior density evaluated at a null value. Hypothesis *H*_1_ and *H*_3_ contain both equality and inequality comparisons and thus has both prior and posterior densities and probabilities. The Bayes factor for *H*_1_ and *H*_3_ against *H*_*u*_ can thus be calculated as *B*_1*u*_ = 4.398 0.075 = 58.265 and *B*_3*u*_ = 0.002 0.055 = 0.036 (see column B(t,u)). The posterior hypothesis probabilities are calculated using Eq. 13 by setting equal prior probabilities, i.e., $\text {Pr}(H_{t}|\textbf {y})=\frac {B_{tu}}{{\sum }_{t^{\prime }} B_{t^{\prime }u}}$, yielding, for example, Pr(*H*_1_|**y**) = 58.265 58.265 + 32.525 + 0.036 + 0.357 = 0.639 (as indicated in column PP(t)).

If **R**_*I*_ is not of full row rank, the posterior and prior that the inequality constraints hold are computed as the proportion of draws from unconstrained Student’s *t* distributions. Under these circumstances, there will be a, typically small, numerical Monte Carlo error. The 90% credibility intervals of the numerical estimate of the Bayes factors of the hypotheses against the unconstrained hypothesis can be obtained by calling

result\InEq{$BFu_{C}I## B(t,u) lb. (5## H1 58.265 58.169 58.360## H2 32.525 32.152 32.910## H3 0.036 NA NA## Hc 0.357 0.356 0.358

where B(t,u) is the Bayes factor of hypothesis *t* against the unconstrained (*u*), lb. (5%) is the lower bound of the 90% credibility interval estimate of the Bayes factor and ub. (95%) is the upper bound. Credibility intervals are only printed when the computed Bayes factors have numerical errors. If the user finds the Monte Carlo error to be too large, they can increase the number of draws from the Student’s *t* distributions by adjusting the input value for the mcrep argument (default 10^6^ draws).

## Discussion

The paper presented a new Bayes factor test for evaluating hypotheses on the relative effects in a linear regression model. The proposed testing procedure has several useful properties such as its flexibility to test multiple equality and/or order constrained hypotheses directly against each other, its intuitive interpretation as a measure of the relative evidence in the data between the hypotheses, and its fast computation. Moreover, no prior information needs to be manually specified about the expected magnitude of the effects before observing the data. Instead, a default procedure is employed where a minimal fraction of the data is used for default prior specification and the remaining fraction is used for hypothesis testing. A consequence of this choice is that the statistical evidence cannot be updated using Bayes’ theorem when observing new data. This is common in default Bayes factors (e.g., O’Hagan, 1997; Berger & Pericchi, 2004). Instead, the statistical evidence needs to be recomputed when new data are observed. This, however, is not a practical problem because of the fast computation of the default Bayes factor due to its analytic expression.

Furthermore, the readily available lmhyp-package can easily be used in combination with the popular lm-package for linear regression analysis. The new method will allow researchers to perform default Bayesian exploratory analyses about the presence of a positive, negative, or zero effect and to perform default Bayesian confirmatory analyses where specific relationships are expected between the regression effects which can be translated to equality and order constraints. The proposed test will therefore be a valuable contribution to the existing literature on Bayes factor tests (e.g., Klugkist, Laudy, & Hoijtink, 2005; Rouder, Speckman, Sun, Morey, & Iverson, 2009; Klugkist, Laudy, & Hoijtink, 2010; van de Schoot et al., 2011; Wetzels & Wagenmakers 2012; Rouder, Morey, Speckman, & Province, 2012; Rouder & Morey 2015; Mulder et al., 2012; Mulder 2014a; Gu, Mulder, Decovic, & Hoijtink, 2014; Mulder 2016; Böing-Messing, van Assen, Hofman, Hoijtink, & Mulder, 2017; Mulder & Fox 2018), which are gradually winning ground as alternatives to classical significance tests in social and behavioral research. Due to this increasing literature, a thorough study about the qualitative and quantitative differences between these Bayes factors is called for. Another useful direction for further research would be to derive Bayesian (interval) estimates under the hypothesis that receives convincing evidence from the data.

## Appendix A: Proof of Lemma 1

A derivation is given for the prior adjusted default Bayes factor for a hypothesis *H*_1_ : **R**_*E*_***β*** = **r**_*E*_,**R**_*I*_***β*** > **r**_*I*_ against an unconstrained hypothesis *H*_*u*_ : ***β*** ∈ *ℝ*^*k*^ in Eq. 9 – 10. Based on the reparameterization ***ξ*** = [ ***ξ***_*E*_***ξ***_*I*_ ] = [ **R**_*E*_**D** ]***β*** in Eq. 5, the hypothesis is equivalent to *H*_1_ : ***ξ***_*E*_ = **r**_*E*_,**R**~_*I*_***ξ***_*I*_ > **r**~_*I*_ against an unconstrained hypothesis *H*_*u*_ : ***ξ*** ∈ *ℝ*^*k*^. The marginal likelihood under the constrained hypothesis *H*_1_ is defined as in the fractional Bayes factor (O’Hagan, 1995) with the exception that we integrate over an adjusted integration region (Mulder, 2014b; Böing-Messing et al., 2017). This adjustment ensures that the implicit default prior is centered on the boundary of the constrained space. The marginal likelihood under *H*_1_ is defined by

19 $$ \begin{array}{@{}rcl@{}} p_{1}(\textbf{y},b) = \frac{\iint_{\tilde{\textbf{R}}_{I}\boldsymbol{\xi}_{I}>\tilde{\textbf{r}}_{I}} p(\textbf{y}|\boldsymbol{\xi}_{E} = \textbf{r}_{E},\boldsymbol{\xi}_{I},\sigma^{2}){\pi_{u}^{N}}(\boldsymbol{\xi}_{I},\sigma^{2})d\boldsymbol{\xi}_{I} d\sigma^{2}} {\iint_{\tilde{\textbf{R}}_{I}\boldsymbol{\xi}_{I}>\textbf{r}^{*}_{I}} p(\textbf{y}|\boldsymbol{\xi}_{E} = \hat{\boldsymbol{\xi}}_{E},\boldsymbol{\xi}_{I},\sigma^{2})^{b}{\pi_{u}^{N}}(\boldsymbol{\xi}_{I},\sigma^{2})d\boldsymbol{\xi}_{I} d\sigma^{2}}. \end{array} $$

As can be seen, the adjustment implies that in the denominator the fraction of the likelihood is evaluated at ***ξ*^**_*E*_ instead of **r**_*E*_ and the integration region equals

$$ \tilde{\textbf{R}}_{I}(\boldsymbol{\xi}_{I}-\hat{\boldsymbol{\xi}}_{I}+{\boldsymbol{\mu}_{I}^{0}})>\tilde{\textbf{r}}_{I} \Leftrightarrow \tilde{\textbf{R}}_{I}\boldsymbol{\xi}_{I}>\tilde{\textbf{R}}_{I}\hat{\boldsymbol{\xi}}_{I}=\textbf{r}_{I}^{*}, $$

because **R**~_*I*_***μ****I*0 = **r**~_*I*_, instead of **R**~_*I*_***ξ***_*I*_ > **r**~_*I*_. Note that ***ξ*^**_*I*_ = **D*****β*^**. This adjustment of the fractional Bayes factor ensures that the proposed default Bayes factor is computed using an implicit default prior that is centered on the boundary of the constrained space, following Jeffreys’ heuristic argument (see Mulder 2014b, for a more comprehensive motivation). Furthermore, this ensures that the complexity of an order-constrained hypothesis is properly incorporated in the Bayes factor (Mulder, 2014a). The marginal likelihood under *H*_*u*_ is defined by

20 $$ \begin{array}{@{}rcl@{}} p_{u}(\textbf{y},b) = \frac{\iiint p(\textbf{y}|\boldsymbol{\xi}_{E},\boldsymbol{\xi}_{I},\sigma^{2}){\pi_{u}^{N}}(\boldsymbol{\xi}_{E},\boldsymbol{\xi}_{I},\sigma^{2})d\boldsymbol{\xi}_{E} d\boldsymbol{\xi}_{I} d\sigma^{2}} {\iiint p(\textbf{y}|\boldsymbol{\xi}_{E},\boldsymbol{\xi}_{I},\sigma^{2})^{b}{\pi_{u}^{N}}(\boldsymbol{\xi}_{E},\boldsymbol{\xi}_{I},\sigma^{2})d\boldsymbol{\xi}_{E} d\boldsymbol{\xi}_{I} d\sigma^{2}}. \end{array} $$

The fraction *b* will be set to *k*+ 1*n* because *k* + 1 observations are needs to obtain a finite marginal likelihood when using a noninformative prior *π**u**N*(***ξ***_*E*_,***ξ***_*I*_,*σ*^2^) = *σ*^− 2^ under *H*_*u*_.

The default Bayes factor is then given by

21 $$ \begin{array}{@{}rcl@{}} B_{1u,b} &=& \frac{p_{1}(\textbf{y},b)}{p_{u}(\textbf{y},b)} = \frac{\iint_{\tilde{\textbf{R}}_{I}\boldsymbol{\xi}_{I}>\tilde{\textbf{r}}_{I}} p(\textbf{y}|\boldsymbol{\xi}_{E}=\textbf{r}_{E},\boldsymbol{\xi}_{I},\sigma^{2}){\pi_{u}^{N}}(\boldsymbol{\xi}_{I},\sigma^{2})d\boldsymbol{\xi}_{I} d\sigma^{2}} {\iint_{\tilde{\textbf{R}}_{I}\boldsymbol{\xi}_{I}>\textbf{r}^{*}_{I}} p(\textbf{y}|\boldsymbol{\xi}_{E}=\hat{\boldsymbol{\xi}}_{E},\boldsymbol{\xi}_{I},\sigma^{2})^{b}{\pi_{u}^{N}}(\boldsymbol{\xi}_{I},\sigma^{2})d\boldsymbol{\xi}_{I} d\sigma^{2}}\text{\Huge{/}} \\ && \frac{\iiint p(\textbf{y}|\boldsymbol{\xi}_{E},\boldsymbol{\xi}_{I},\sigma^{2}){\pi_{u}^{N}}(\boldsymbol{\xi}_{E},\boldsymbol{\xi}_{I},\sigma^{2})d\boldsymbol{\xi}_{E} d\boldsymbol{\xi}_{I} d\sigma^{2}} {\iiint p(\textbf{y}|\boldsymbol{\xi}_{E},\boldsymbol{\xi}_{I},\sigma^{2})^{b}{\pi_{u}^{N}}(\boldsymbol{\xi}_{E},\boldsymbol{\xi}_{I},\sigma^{2})d\boldsymbol{\xi}_{E} d\boldsymbol{\xi}_{I} d\sigma^{2}}\\ &=& \iint_{\tilde{\textbf{R}}_{I}\boldsymbol{\xi}_{I}>\tilde{\textbf{r}}_{I}}\frac{ p(\textbf{y}|\boldsymbol{\xi}_{E}=\textbf{r}_{E},\boldsymbol{\xi}_{I},\sigma^{2}){\pi_{u}^{N}}(\boldsymbol{\xi}_{I},\sigma^{2})} {\iiint p(\textbf{y}|\boldsymbol{\xi}_{E},\boldsymbol{\xi}_{I},\sigma^{2}){\pi_{u}^{N}}(\boldsymbol{\xi}_{E},\boldsymbol{\xi}_{I},\sigma^{2})d\boldsymbol{\xi}_{E} d\boldsymbol{\xi}_{I} d\sigma^{2}}d\boldsymbol{\xi}_{I} d\sigma^{2} \text{\Huge{/}} \\ && \iint_{\tilde{\textbf{R}}_{I}\boldsymbol{\xi}_{I}>\textbf{r}^{*}_{I}}\frac { p(\textbf{y}|\boldsymbol{\xi}_{E}=\hat{\boldsymbol{\xi}}_{E},\boldsymbol{\xi}_{I},\sigma^{2})^{b}{\pi_{u}^{N}}(\boldsymbol{\xi}_{I},\sigma^{2})} {\iiint p(\textbf{y}|\boldsymbol{\xi}_{E},\boldsymbol{\xi}_{I},\sigma^{2})^{b}{\pi_{u}^{N}}(\boldsymbol{\xi}_{E},\boldsymbol{\xi}_{I},\sigma^{2})d\boldsymbol{\xi}_{E} d\boldsymbol{\xi}_{I} d\sigma^{2}} d\boldsymbol{\xi}_{I} d\sigma^{2}\\ &=& \iint_{\tilde{\textbf{R}}_{I}\boldsymbol{\xi}_{I}>\tilde{\textbf{r}}_{I}}\pi_{u}(\boldsymbol{\xi}_{E}=\textbf{r}_{E},\boldsymbol{\xi}_{I},\sigma^{2}|\textbf{y}) d\boldsymbol{\xi}_{I} d\sigma^{2} \text{\Huge{/}} \\ && \iint_{\tilde{\textbf{R}}_{I}\boldsymbol{\xi}_{I}>\textbf{r}^{*}_{I}}\pi_{u}(\boldsymbol{\xi}_{E}=\hat{\boldsymbol{\xi}}_{E},\boldsymbol{\xi}_{I},\sigma^{2}|\textbf{y}^{b}) d\boldsymbol{\xi}_{I} d\sigma^{2} \\ &=& \frac{\text{Pr}(\tilde{\textbf{R}}_{I}\boldsymbol{\xi}_{I}>\tilde{\textbf{r}}_{I}|\textbf{y},\boldsymbol{\xi}_{E}=\textbf{r}_{E})} {\text{Pr}(\tilde{\textbf{R}}_{I}\boldsymbol{\xi}_{I}>\textbf{r}^{*}_{I}|\textbf{y},\boldsymbol{\xi}_{E}=\hat{\boldsymbol{\xi}}_{E})}\times \frac{\pi_{u}(\boldsymbol{\xi}_{E}=\textbf{r}_{E}|\textbf{y})}{\pi_{u}(\boldsymbol{\xi}_{E}=\hat{\boldsymbol{\xi}}_{E}|\textbf{y}^{b})}. \end{array} $$

Furthermore, using standard calculus it can be shown that the marginal posterior for ***β*** for a fraction *b* of the data and a noninformative prior has a Student’s *t* distribution

$$ \begin{array}{@{}rcl@{}} \pi_{u}(\boldsymbol{\beta}|\textbf{y}^{b}) &\propto &\int p(\textbf{y}|\boldsymbol{\beta},\sigma^{2})^{b} {\pi_{u}^{N}}(\boldsymbol{\beta},\sigma^{2}) d\sigma^{2}\\ &\propto& t(\boldsymbol{\beta};\hat{\boldsymbol{\beta}},s^{2}(nb-k)^{-1}(\textbf{X}^{\prime}\textbf{X})^{-1},nb-k), \end{array} $$

and therefore, because ***ξ*** = **T*****β***|**y**^*b*^, it holds that

$$ \begin{array}{@{}rcl@{}} \pi_{u}(\boldsymbol{\xi}|\textbf{y}^{b}) = t(\boldsymbol{\xi};\hat{\boldsymbol{\xi}},s^{2}(nb-k)^{-1}\textbf{T}(\textbf{X}^{\prime}\textbf{X})^{-1}\textbf{T}^{\prime},nb-k), \end{array} $$

where **T** = [ **R**_*E*_**D** ], ***ξ*^** = (***ξ*^***E*′,***ξ*^***I*′)^′^ with ***ξ*^**_*E*_ = **R**_*E*_***β*^** and ***ξ*^**_*I*_ = **D*****β*^**, and *t*(***ξ***;**m**,**K**,*ν*) denotes a Student’s *t* distribution for ***ξ*** with location parameters **m**, scale matrix **K**, and *ν* degrees of freedom. Then it is well known that the marginal distribution of ***ξ***_*E*_ and the conditional distribution of ***ξ***_*I*_|***ξ***_*E*_ also have Student’s *t* distributions (e.g., Press 2005) given by

22 $$ \begin{array}{@{}rcl@{}} \boldsymbol{\xi}_{E}|\textbf{y}^{b} \!& \sim &\! t(\hat{\boldsymbol{\xi}}_{E},s^{2}(nb - k)^{-1}\textbf{R}_{E}(\textbf{X}^{\prime}\textbf{X})^{-1}\textbf{R}^{\prime}_{E},nb - k) \end{array} $$

23 $$ \begin{array}{@{}rcl@{}} \boldsymbol{\xi}_{I}|\boldsymbol{\xi}_{E},\textbf{y}^{b} \!& \sim &\! t(\textbf{m}_{I|E},\textbf{K}_{I|E},\nu), \end{array} $$

with

$$ \begin{array}{@{}rcl@{}} \textbf{m}_{I|E} & = & \textbf{D}\hat{\boldsymbol{\beta}}+s^{-2}(nb-k)\textbf{D}(\textbf{X}^{\prime}\textbf{X})^{-1}\textbf{R}_{E}^{\prime}(\textbf{R}_{E}(\textbf{X}\textbf{X})^{-1}\textbf{R}_{E})^{-1}(\boldsymbol{\xi}_{E}-\hat{\boldsymbol{\xi}}_{E})\\ \textbf{K}_{I|E} & = & \frac{nb-k + s^{-2}(nb-k)(\boldsymbol{\xi}_{E}-\hat{\boldsymbol{\xi}}_{E})'(\textbf{R}_{E}^{\prime}(\textbf{X}^{\prime}\textbf{X})^{-1}\textbf{R}_{E})^{-1}(\boldsymbol{\xi}_{E}-\hat{\boldsymbol{\xi}}_{E})}{nb-k+q^{E}}\\ &&(s^{2}(nb-k)^{-1}\textbf{D}(\textbf{X}^{\prime}\textbf{X})^{-1}\textbf{D}^{\prime}-\\ && s^{2}(nb-k)^{-1} \textbf{D}(\textbf{X}^{\prime}\textbf{X})^{-1}\textbf{R}_{E}^{\prime}(\textbf{R}_{E}(\textbf{X}^{\prime}\textbf{X})^{-1}\textbf{R}_{E}^{\prime})^{-1}\textbf{R}_{E}(\textbf{X}^{\prime}\textbf{X})^{-1}\textbf{D}). \end{array} $$

Thus, when plugging in *b* = 1 and ***ξ***_*E*_ = **r**_*E*_ in Eqs. 22 and 23, and then in Eq. 21, gives the numerators in Eqs. 9 and 10, and plugging in *b* = *k*+ 1 *n* and ***ξ***_*E*_ = ***ξ*^**_*E*_ in Eqs. 22 and 23, and then in Eq. 21, gives the denominators in Eqs. 9 and 10, which completes the proof.

## Appendix B: Example analysis for Fig. 1

# consider a regression model with two predictors:# y_{i} = beta_{0} + beta_{1} * x1_{i} + beta_{2} * x2_{i} + errorlibrary(lmhyp)n <- 20 #sample sizeX <- mvtnorm::rmvnorm(n,sigma=diag(3))# For this example we transform X to get exact independent# predictor variables and errors. X <- X - rep(1,n)X <- Xerrors <- X[,3] #a population variance of 1X <- X[,1:2]beta <- c(.7,.03) #data generating regression effectsy <- 1 + Xdf1 <- data.frame(y=y,x1=X[,1],x2=X[,2])fit1 <- lm(y~x1+x2,df1)test1 <- test_{h}yp(fit1,"x1=x2=0;(x1,x2)>0;x1>x2=0")test1 #get posterior probabilities test1$}BF_matrix #get Bayes factorstest1\(BF_{c}omputation #get details on the computationstest1\)BFu_CI #get 90

## Footnotes

1 The proper default prior in Eq. 8 is obtained by updating the noninformative improper (independence) Jeffreys’ prior, *π*^*N*^(***β***,*σ*^2^) ∝ *σ*^− 2^, with a fraction *b* of the data: *π*_*t*_(***β***,*σ*^2^|**y**^*b*^) ∝ *π*^*N*^(***β***,*σ*^2^)*f*_*t*_(**y**|***β***,*σ*^2^)^*b*^ (Gilks, 1995).

2 Updating the noninformative Jeffreys prior *π*^*N*^(***β***,*σ*) ∝ *σ*^− 2^ with a sample of *k* + 1 observations yields a proper marginal distribution for ***β*** having a multivariate Student’s *t* distribution with 1 degree of freedom, which is equivalent to a multivariate Cauchy distribution.

3 Run ‘devtools::install_github("jaeoc/lmhyp")’ in R to install the package.

4 The R-script used to produce the results in this section is available at https://osf.io/g8c9p/
